# Ship Fever

**Published:** 1847-08

**Authors:** 


*Ship Fever.*—The following report of the committee appointed by the Academy of Medicine, N. Y., to investigate the fever prevailing so extensively among the newly arrived immigrants, we find in the columns of the New York Herald, it having been communicated to the common council of the city, and ordered to be printed. We shall look with impatience for the final report of the committee, and, so soon as received, shall lay it before our readers. —Editor Buff. Med. Jour.

At the last meeting of the Academy of Medicine, the following report on the subject of ship fever, which has been the cause of so much excitement and alarm among our citizens, was read by Dr. Stewart, in behalf of a committee appointed at a previous meeting to investigate the subject.

NEW-YORK, July 7, 1847.

The committee appointed at the last meeting of the Academy, to investigate the subject of the prevailing typhus, typhoid, or ship fever, respectfully Report,

That in accordance with their instructions, they have visited and inspected all the public hospitals and private institutions in the vicinity of the city into which fever cases have been received; and the result of their investigations has been so far satisfactory, as to have enabled them to obtain a vast amount of valuable general and statistical information in regard to the subject upon which they are required to report; which will place it in their power, at some future period—when sufficient time shall have been allowed to collect and arrange all the data upon which their report will be based—to present what they conceive will be an interesting and valuable record of medical experience, in regard

1st. To the origin, causes, and mode of propagation of the fever of the present season.

2d. To its distinctive characters.

3d. To its autopsic phenomena.

4th. To its statistics, and

5th. To the course of treatment which has been attended with the most satisfactory results.

Sub-committees have been appointed to examine into each of these departments of the general subject, and a correspondence has been opened with the health officers of various seaports on our continent, at which the disease is prevailing.

As a considerable time must elapse, however. before all the expected returns can be received, and a final and satisfactory report rendered your committee have deemed it expedient, in view of the state of public alarm in regard to this fever, to submit to you at the present time, some few general facts and conclusions in connection with the subject ,which, if allowed to go forth with the sanction and approval of the Academy, will be calculated to remove from the public mind all fear in relation to the danger to be apprehended, either by citizens or strangers, from this cause.

That there has been during the past three months an unusual number of cases of typhus, or ship fever, in our public and private hospitals,is undoubtedly true; it has been confined, however, almost entirely to emigrants, and particularly, to those arriving from Ireland, to whose unhappy condition at home, and the criminal negligence of those engaged in transporting them to our shores, may be attributed the vast amount of suffering and sickness to which they have been subjected.

The records of the Commissioners of Health show that no less a number of steerage or emigrant passengers than 84,218 arrived at this port during the first six months of the present year, and of these 74,428 have been landed since the first of April; giving a monthly average since the latter date of nearly 25,000.

This immense increase of emigration is alone sufficient to account for a large increase in the number of cases of a disease which always prevails with us at the period of the year when emigrants are arriving. In addition to this, however, other causes, already alluded to, have had a material influence in causing the sickness of the present season.

It is now a well established fact, that typhus, ship, or jail fever is capable of being produced at any time when a large number of persons are congregated together in a confined space, and deprived of the means of cleanliness, pure air, and proper nourishment, and as most of these causes have existed to an unparalleled extent in the case of emigrants of the present season, it is rather a matter of surprise that so small a number, comparatively speaking, should have suffered from it. In the case of the Irish paupers introduced amongst us, all these causes have been in full operation; previous to embarkation they had been for a long period in a state of utter destitution—but little short of actual starvation. They have been taken on board ship in a filthy condition, and in most cases were unprovided with a single change of clothing. Numbers of them, to the extent, in some instances of more than five hundred, have been received into the steerage of one vessel, and their condition at sea has sometime been most deplorable. In a British vessel, (the ship Ceylon,) which your Committee were, through the politeness of the health officer, and his deputy, Dr. Harcourt, enabled to visit and inspect on her arrival, we found 257 passengers huddled together in the steerage, which was in a most foul state. This vessel had lost as many as 30 of her passengers, previous to her arrival, and 115 were then so ill as to render it necessary to land them at the quarantine hospital.

At other ports on this continent, vessels have arrived in a still worse condition. The ship Loosthauk, from Liverpool to Quebec, had to put into Chatham in distress. She had on leaving England, 349 steerage passengers, of whom 117 had died on the passage, and only 20 persons on board had escaped sickness. Five emigrant vessels arrived at Quebec, about the middle of the last month, which had lost at sea no less than 275 of their passengers—an average of 55 for each one of them.

The returns made to our health officer, at Staten Island, by captains of vessels arriving here, show an aggegrate of 947 deaths at sea on board of vessels coming from European Ports; and three-fourths of the number addmitted into the quarantine hospital (most of them Irish) have been taken from British vessels.

These facts prove conclusively what is the cause to which we are to attribute the increase of ship fever during the present season.

Notwithstanding this great increase, however, we think that no danger need be apprehended by our citizens. But few of the causes, productive of the disease, exist among us; and no apprehension need be felt of its becoming epidemic, so long as due attention is paid to cleanliness and ventilation.

The disease has, so far, been exclusively confined to emigrants, and those in constant attendance upon them—such as physicians, medical students, and nurses. We have been unable to collect the particulars of more than two or three cases, which were not traceable directly to intercourse with those laboring under the disease, and who had lately arrived from sea. Although hundreds of cases have been congregated in our public and private hospitals, no person living in their vicinity has been attacked. None have suffered but those who, as has been already stated, are in constant intercourse with, and daily attendance upon, the sick.

The bills of mortality, too, show that the disease has been confined almost entirely to the hospitals.

From the City inspector's returns, we gather that the whole number of deaths, in this city, from typhoid and typhus (ship) fever, was, from the 2d day of January to the 26th of June, inclusive, 570

Of these there died—

| | | |
|---|---|---|
| At the Bellevus Hospital . . . . . . . | 260 | |
| " Children's Hospital on Blackwell's Island | 18 | |
| " Penitentiary Hospital . . . . . . | 6 | |
| " City Hospital . . . . . . . . | 62 | |
| " Private Hospitals at Bloomingdale and Harlem (whole mortality 201, of which as these hospitals are for sick emigrants exclusively, it is fair to presume the greater part were of ship fever), say two-thirds, or . | 134 | |
| Total of deaths in hospitals . . . . . . . . | 480 | |
| Leaving for all other public institutions and the city generally, only . . . . . . . . . . . | 90 | |
| | | 570 |

This is a mortality so trifling, when it is borne in mind that it occurs in a population of more than 400,000, and embraces a period of six months, that it affords no ground whatever for apprehension.

In addition to all this moreover, ample provision has now been made by the Commissioners of Emigration to provide for the accommodation of sick emigrants without the city precincts.

From the foregoing facts, and other information in their possession, your committee feel themselves fully justified in presenting for your consideration and adoption the following conclusions:—

1. That although there has been a decided increase in the number of cases of typhus, or ship fever, in our city during the present season, as compared with other seasons, such increase is only in proportion to the increased emigration of the present year, and the bad condition of the emigrants.

2. That the disease is confined almost exclusively to emigrants, and those who are in direct and constant attendance upon such of them as are sick.

3. That no danger need be apprehended of the disease becoming epidemic; and that, with a due regard to cleanliness and ventilation, our citizens have no cause whatever, for alarm on the subject. All of which is respectfully submitted.

Ordered to be printed.

---

*Ship Fever in Canada.*—We quote the following from the British American Journal of Medical and Physical Science.

"Up to the present moment, 32,338 emigrants have landed on our shores; and of this number, exclusive of those who have died on the passage, and are sick in this city, Quebec, and the intermediate places, (and of whom we have no record, because not entering the hospitals,) upwards of 5000 have been known to have been ill—being about one-sixth of the whole number. The medical staff at the Quarantine, and at the Emigrant Sheds in the vicinity of this city, have received additions to their numbers; but are yet insufficient to meet the exigency. The medical officers in charge are worn out and harrassed with their ardous and unceasing labors. The mortality is appalling. At the Quarantine establishment at Grosse Isle, 2,796 cases, of which more than four-fifths were cases of fever, have been treated between the 8th of May 19th June. Of this number 565 have died, being at the rate of 1 in every 4.9 cases, or 20.4 per cent. At the Emigrant Hospital, in the neighborhood of this city, between the 13th and 28th of June, 1420 cases, all of fever, were admitted—the deaths during the same period 331, affording a greater ratio of mortality, the rate being 1 to every 4.2 cases, or 23,8 per cent. We have not been enabled to arrive at any satisfactory conclusion with reference to the Marine Hospital at Quebec, no return having been made to the Provincial Secretary's Office anteriorily to the 20th of June. The weekly return during the period between the 20th and 26th June, gave a total number of 859 cases; of which 394 were cases of fever, and 24 of dysentery. The mortality during the same period was 27. Assuming that this mortality was met with exclusively among these cases, it would form a rate of 1 to every 15.4, or 6.4 per cent, a ratio so disproportionate to what has been observed at the other stations, that we can place no reliance upon it, as indication of the character or type of the cases admitted. We are enabled to speak more positively and with greater certainty, with reference to the Montreal General Hospital. From the 28th May to the 28th June, 298 sick emigrants were admitted. Of these 143 were cases of typhus, 18 were cases of diarrhœa, and 97 of common continued fever. The mortality among the typhus cases, every one of which was of the petechial type, was 1 to 4.9, or 20.4 per cent. Of the cases of simple continued fever and diarrhœa, one only, respectively, died; while estimating the mortality upon the general number of admissions, as we have done in the other institutions, we find it to have been for the same period, 1 to every 7.7 cases, or 12.9 per cent., the total admissions having been 324, and the deaths 42.

We regret that our data a[illegible] so imperfect. The returns from the institutions at Grosse Isle, [illegible]e Marine Hospital, and the Emigrant Sheds in this city, affo[illegible] but general results. The deaths are recorded, but without specifying the particular diseases under which the mortality occurred. For general purposes, they may be turned to advantage, but for special ends, such as the ratio of the mortality of a particular disease, they are valueless. The return of the Montreal General Hospital may be assumed as a fair criterion of the average mortality of the ship fever, even under its most favorable chances of treatment, and portrays its malignant character, and its fatality, in a manner which cannot be misinterpreted.

We do not wish to be considered as alarmists, nor are we writing for the purpose of engendering any unnecessary or groundless apprehensions. The disease is an eminently contagious one; and the records of private practice, both in this city and Quebec, (as we have been informed,) proclaim its steady progress among the inhabitants. To be forewarned is to be forearmed. Let the strictest cleanliness prevail everywhere. Let the Boards of Health be active and energetic in their duties, and let the civic authorities pay immediate attention to their reports, and carry out their suggestions with vigorous determination, laying all pecuniary considerations aside. Public health is public wealth; by paying due regard to the former, the latter is certain to follow; and the funds of the city are much better employed, and will produce a greater and more satisfactory return by the mode indicated, than by all the embellishments which the same amount of money might command. We think that the emigrants, under existing circumstances, should be debarred all communication with the citizens.

